# Fluctuation between Powerlessness and Sense of Meaning - A Qualitative Study of Health Care Professionals’ Experiences of Providing Health Care to Older Adults with Long-Term Musculoskeletal Pain

**DOI:** 10.1186/s12877-015-0088-y

**Published:** 2015-08-04

**Authors:** Mia Berglund, Kristina Nässén, Catharina Gillsjö

**Affiliations:** 1 School of Health and Education, Health and Learning Research Centre: Aging and Long-Term Health Problems, University of Skövde, Skövde, Sweden; 2 Academy of Care, Working Life and Social Welfare, University of Borås, Borås, Sweden; 3 College of Nursing, University of Rhode Island, Kingston, USA

**Keywords:** Health care providers, Experiences, Older adults, Long-term musculoskeletal pain, Home

## Abstract

**Background:**

There is an increasing number of older adults living with long-term musculoskeletal pain and related disabilities. These problems are frequently unrecognized, underreported, and inadequately treated. Since many older adults desire to remain at home for as long as possible, it is important that individualized and holistically tailored care is provided in these settings. However, there is a complexity in providing care in this context. The aim of this study was to describe health care professionals’ experiences of providing health care to older adults living with long-term musculoskeletal pain at home.

**Methods:**

The phenomenon, “To provide health care to older adults living with long-term musculoskeletal pain at home”, was studied using reflective lifeworld research (RLR) which is based on phenomenological epistemology. Ten health care providers (nurse, physiotherapists, and occupational therapists) were interviewed and data was analysed.

**Results:**

The health care professional’s emotions fluctuated between powerlessness and meaningfulness. Needs, opportunities, understanding and respect had to be balanced in the striving to do good in the provision of health care in differing situations. Caring for older adults with long-term pain required courage to remain in the encounter despite feelings of insecurity and uncertainty about the direction of the dialogue. The essence of caring for older adults with long-term pain consisted of the following constituents: Sense of powerlessness; striving to provide good health care; and understanding and respect.

**Conclusions:**

The findings indicated that the health care professionals strived to do good and to provide health care that was holistic and sensitive to the older adults’ needs. A significant sense of powerlessness in the situation was experienced by the health care professionals. These findings address and support the need to develop methods that can be used to guide health care providers who support older adults in the context of their homes.

## Background

It is well known that the population of older adults is rapidly increasing worldwide [1]. Increasing age is often associated with complex health problems, often in combination with long-term musculoskeletal pain. In contrast to other types of pain, musculoskeletal pain tends to increase with age [2, 3]. The prevalence rate is estimated to be as high as 60 % among community dwelling older adults [3–5]. Long-term musculoskeletal pain is recognized as a main cause of disabilities among older adults and has a significant impact on quality in life [5–9]. Despite increasing frailty [10] and dependence, many older adults desire to remain at home for as long as possible and to receive help, if needed, from home health care providers [11–15]. This direction in provision of health care to older adults is supported by health care and social policies [16, 17]. The literature on musculoskeletal pain to date has mainly focused on prevalence, underlying cause, management and associated disabilities [18]. There is limited literature focusing on older adults’ experiences of living with long-term musculoskeletal pain, but dissatisfaction with care is an issue [19, 20]. Far less attention is provided in the literature on the providers’ experiences of caring for these older adults. This study addresses the need to explore health care professional’s experiences of providing health care to older adults living with long-term musculoskeletal pain at home.

### Older adults with long-term pain

Regardless of the frequency of musculoskeletal pain and its impact in older adults’ lives, researchers continue to report that this type of pain, like long-term pain in general, is frequently unrecognized, underreported, and inadequately treated among older adults [21–24]. One explanation might be that pain frequently is viewed by older adults, relatives and health care providers as a natural part of the process of aging [25, 26]. There is growing recognition, however, that health care providers’ lack a high degree of knowledge and engagement in relation to older adults’ pain [26, 27]. Research also indicates a tendency toward stoic attitudes that are associated with reticence to report pain in this population [28, 29]. This reticence can be explained in part by older adults’ overall orientation in life to continue daily life despite pain. Living with pain on a daily basis was shown to be less about pain management than about how to endure the pain. A major commonality in this act of enduring was that older adults felt forced into learning to live with pain on their own [19]. Eccleston and Crombez [30] described long-term pain as feared, inescapable and threatening to human beings in their environment. The authors highlighted the constant interplay between the human being, the characteristics of the pain, and the demands of the environment as a threat to the sense of being a whole person.

### Health care providers’ attitudes

Health care providers’ attitudes can be challenging, since there is a prevailing opinion that providing health care to older adults is “basic” and that little knowledge or skill is required. Attitudes influence providers’ quality of care and the older adults’ dignity and autonomy [31]. In the existing literature there is a history of negative attitudes among professionals towards providing health care to older adults [32, 33]. Kane [34] found an existence of these attitudes when healthcare professionals perceived that clinical interventions for older adults were not worth the investment. Gallagher, Bennett and Halford [31] found that level of education was an important predictor for attitudes. Lyons, Dunson-Strane and Sherman [35] argued the need for education to increase knowledge and understanding about older adults’ needs in their context in order to bring joy into caring within the older adults’ context.

### Theoretical framework

The sense of being a whole person can be viewed in light of the lifeworld perspective in which the physical, mental and existential dimensions are intertwined [36–38] and the person is understood as a lived body [38]. The perspective of lifeworld can be used in lifeworld-led care which includes three dimensions: a philosophy of the person, a focus on well-being and not the illness per see, and a philosophy of care that addresses this core [39]. Lifeworld-led care [39] provides a direction for care and practice that is intrinsically and positively health focused. Todres, Galvin and Dahlberg [40] explored “Caring for Insiders” as a care where health care providers are aware of “insiderness” that recedes from the view of the other and can never be fully grasped. Striving to reach towards “insiderness” is complex. It requires the use of self through “reflective openheartness” and knowledge of lifeworld-led care.

A significant prerequisite and critical aspect in seeing the patient as a whole in the provision of health care is the concept of “knowing the patient” [41–43]. This concept refers to gaining an in-depth understanding of a person in a specific context, including background and way of living [43, 44]. This is fundamental as health care providers act to help patients maintain their integrity and continue life despite health problems [45]. Knowing the patient takes time and is related to the situation and context. It requires knowledge of the person’s history, how the person lives, what gives meaning in life and how the person reacts and acts in various situations [42, 43, 45, 46].

The interaction in the provision of care within the perspectives of “lifeworld-led care” and “knowing the patient” is closely linked with lived experience [39, 42, 43, 46] which is essential in the provision of holistic health care. The literature is limited on how to develop and maintain this type of interaction [47]. There is, however, an increasing interest for person-centered care [48], in which a partnership evolves and where the person’s life situation comes to the forefront, and not the disease itself. Furthermore, an awareness of moral concerns and protection of patient agency is important in the context [49]. This raises the need to focus on the question of “how to do” instead of “what to do” in the act of nursing in regard to moral issues [50]. Furthermore, Liaschenko [50] highlighted the challenge for nurses to “sharpen the gaze of nursing” (p. 25) which involves a focus and courage to help patients to live their lives in their context instead of focusing on disease as in the biomedical model. This need is supported by Kim [44, 51] whose model and concept of “human living” encompasses the environment, interactions and phenomena in patients’ lives, all of which can be used to help patients live their lives with their health problems. The need for providing health care that is more sensitive to place and more fully based on individuals in their context has been addressed by other scholars and practitioners [49, 50, 52–54]. Further understanding is needed in order to develop individualized and holistically tailored care in the older adult’s home, care that takes into account the whole person in the situation, while still preserving the home as a private arena.

### Statement of problem

Providing health care to older adults is often associated with negative attitudes and a low priority as a potential problem in society [31–34]. There is an increasing number of older adults living at home with health problems such as pain. Although older adults desire to remain at home as long as possible, they are not fully satisfied with the care provided. Clearly, it is a challenge for health care providers and society at large to preserve and promote health, well-being, and overall quality in life for the increasing number of frail older adults with complex needs for care at home. This requires providing health care that is individual, holistic and sensitive, as well as methods that can be used to guide and support both older adults and health care providers (c.f. [18, 55]). The current need is to gain an understanding of health care professionals’ experiences of caring for older adults at home.

### Aim

The aim was to describe health care professionals’ experiences of providing health care to older adults living with long-term musculoskeletal pain at home.

## Methods

The ontological and epistemological suppositions in this article are based on lifeworld theory [36–38]. The phenomenon, “Provision of health care to older adults living with long-term musculoskeletal pain at home” has been studied using the approach of Reflective Lifeworld Research (RLR) which is based on phenomenological epistemology [56] and described in Giorgi’s phenomenological approach [57]. In the RLR approach, openness and orientation towards the phenomenon are used to guide the collection and analysis of data. This openness is achieved through a conscious and deliberate understanding that “bridles” the researcher’s pre-understanding in relation to the studied phenomenon [58]. The bridling is a conscious approach used to maintain a scientific, reflective and sensitive attitude with the aim of increasing understanding of the phenomenon.

### Data collection and participants

Data were collected through qualitative interviews with registered health care professionals (nurse, physiotherapist, occupational therapist) in the context of home health care in three communities in the western region of Sweden. The term health care professional was used in this study to label the participants in the study. The communities are responsible for provision of health care to older adults above 65 years in assisted living facilities by Swedish legislation. They have the opportunity to transfer the provision of home health care in ordinary homes from county councils to communities to offer an integrated and coherent care that includes both social services and health care. This transfer of care has been done in the three participating communities. The communities’ responsibility related to health care include the registered professions nurses, physiotherapists, occupational therapists and psychologists but exclude physicians The responsibility in the communities is to integrate social care and health care with a focus on autonomy and provision of holistic care to increase quality in life and safety in the older adults’ chosen living environment [59–61].

The chosen health professionals worked in close collaboration in the provision of health care to older adults in their homes. The heads of the community-based healthcare services gave their approval to participate in the study. The head of the unit in each community identified and asked health care professionals that met the inclusion criteria if they were willing to participate. Each health care professional was required to have had at least 3 years of experience in providing health care to the chosen population. Health care providers who consented to participate in the study were contacted by phone by the researcher and a time for the interview was set. Ten health care professionals (eight women and two men) gave their informed consent (nurse *n* = 5, physiotherapists *n* = 3, occupational therapists *n* = 2). Two of the participants were male and eight female in ages ranging from 35 to56 years (mean = 52 years). The participants’ had worked between 5 and 34 years (mean = 19.5) with older adults suffering from pain.

Data were collected through interviews grounded in the RLR approach. The researchers approached the health care professionals with a “bridled” attitude [58] towards their lifeworld so that they would not influence the health care professionals’ descriptions of their experiences of providing health care to older adults’ living with long-term musculoskeletal pain at home. This type of qualitative interview has been described by Dahlberg, Dahlberg and Nyström [56] as a dialogue in which participants reflect upon their experiences to deepen the understanding of the phenomenon. The researchers’ (MB, KN, CG) approach during the interviews was to facilitate an open and allowing atmosphere in the dialogue to encourage the participants’ reflections. The reflective process was initialized by the question, “Would you please tell me about your experience of providing health care to older adults living with long-term musculoskeletal pain at home?” The participants were asked to further develop their answers and give concrete examples of the experiences. Additional questions arose out of the descriptions and were used to orient the attention towards the phenomenon in order to gain a deeper understanding. The interviews were audio-recorded and transcribed verbatim.

### Data analysis

The researchers’ analysis of the interviews followed the RLR approach [56]. The analysis was directed towards discovering patterns and nuances of qualitative meanings that emerged from the transcribed text. The analysis was characterised by openness and sensitiveness in an intensive dialogue with the text. This dialogue vacillated from parts to whole with the aim of deepening the understanding of the phenomenon: “Provision of health care to older adults living with long-term musculoskeletal pain at home”. The researchers’ continuously reflected upon their own understanding and tried out various tentative understandings, while still remaining open and attentive to the text. Demanding and critical questions were present throughout the process of analysis in order to consciously “bridle” the researchers’ pre-understanding and understanding (c.f. [58]). The analysis encompassed movements beyond given conditions and avoidance of linear or causal explanations, which allowed various meanings of the phenomenon to emerge. After reading the text as a whole several times, meaning units (a word, a sentence or a longer section of text) were drawn from the text. The next step in the analysis was to build clusters with similar meaning units. Initially, there were many clusters created, which were aggregated to a less number of clusters due to similarities. The analysis continued and the essence of the phenomenon could be described and constituents identified. The essence can be understood as the core aspects of a phenomenon on an abstract level whereas the four constituents describe the essence of the phenomenon on a concrete level (c.f. [62]). The essence in this study is presented first in the findings, followed by its constituents and quotes to illuminate the findings.

### Ethical considerations

This study followed the principles outlined in the Declaration of Helsinki [63]. Additionally, it was approved by the Regional Ethical Review Board in Gothenburg (814–13). Furthermore, approvals were given by the heads of the social welfare and home health care services in the communities. The participants were informed, both orally and in writing and gave their informed consent to participate in the study. They were also informed that they could interrupt their participation at any time without explanation and consequences. The data were treated confidentially to protect each participant’s identity.

## Results

Provision of health care to older adults with long-term pain entails a fluctuating emotion between powerlessness and meaningfulness. Needs, opportunities, understanding and respect are balanced in the continuous striving to do good in in providing care. Caring for older adults with long-term pain requires courage to remain in the encounter despite the feeling of insecurity and uncertainty about the direction in the dialogue.

The essence of caring for older adults with long-term pain is composed by the following:

- Sense of powerlessness
- Striving to provide good health care
- Understanding and respect

### Sense of powerlessness

The health care professionals experience a sense of powerlessness in the provision of health care to older adults living with long-term pain at home. This emotion is expressed through feelings of frustration, inadequacy and hopelessness. The provision of health care gives the insight that the pain cannot always be cured. This is expressed as “*Sometimes one experiences a sense of powerlessness when one cannot help them”.* It is acknowledged that many of the older adults have had their pain for a long time and that it has been incorporated as a natural part in life. The state of “*being in pain*” as being expressed by a health care professional has become a condition that is endured in silence which sometimes complicates the health care provider’s ability to find ways to approach older adults’ pain. Several health professionals express that they sometimes feel both angry and sad. The sense of powerlessness manifests itself in situations where health care professionals find it difficult to get the attention needed from other health care providers as physicians and assistant nurses to meet the older adults’ needs. The health care professionals are aware that pain does not need to be a natural part in the process of aging which is expressed in statements as:

“*We address the fact that pain does not need to be a natural part of the aging process…one does not have to accept the pain straight off without trying to do something about it.*”

The sense of powerlessness occurs in situations where health care professionals feel that other health care providers do not listen to or believe in the older adult. The emotion is also grounded in the experience of a prevailing opinion among healthcare providers, older adults and significant others that pain belongs to the aging process. Examples of this sense of powerlessness is described by several health care professionals in relation to situations when they experience that physicians do not listen to them and fail to recognize older adults’ needs for pain relief. They feel that they in their role as the older adults’ primary contact and caregiver have an understanding of the situation and existing needs. Situations like this are stressful and lead to conflicts related to differences in demands and expectations between physicians and older adults. It is described through examples as when a physician’s rigor regarding pain medication contrasts the older adult’s desire to live a decent life with endurable pain. The sense of powerlessness is also related to the older adult’s willingness or unwillingness to comply with the proposed intervention. In addition, the health care professionals experience a difficulty in deciding whether to recommend and motivate older adults to take analgesics or to dissuade them. This difficulty is based on considerations about anticipated side effects and risks associated with the medication such as drowsiness, dizziness, risk of falls, confusion, cognitive loss and constipation in relation to the older adult’s health, wellbeing and daily living.

The sense of powerlessness increases with explicit and implicit expectations from others that the pain should be eliminated. The health care professionals have to face the despair of relatives and their own health care team: *“No one should be in pain, so it is always said … it sounds easy, but I think it’s really difficult”.* In contrast, the health care professionals sometimes find that the older adults themselves seem to be the least despaired in the situation with pain. They have the insight that *“The older adult might have had pain for 25 years”,* as expressed by one health care professional. A complex situation which adds to the sense of powerlessness is when they try to give information but realize that the older adults, significant others and care givers have different perspectives and find it difficult to understand the limited possibility to completely eliminate the pain. The health care professionals try different methods as TENS and daycare to help alleviate the pain as a complement to the prescribed medication. They feel frustrated and sometimes resign when they feel they try everything and nothing works. One health care professional says:

*“…sometimes one becomes a little resigned when one feels that you’ve tried everything and nothing works, then one can feel a frustration together with the patient, one wants so badly to be able to relieve the pain.”*

Health care professionals’ say, that if they have had more time, they might have been able to capture the older adults’ needs and individual situation more thoroughly. They have the insight of how easy it is to focus on the problem and its solution, without reaching the core of the problem in the situation. The health care professionals sometimes become disappointed when the older adult does not want to use the proposed actions to ease the pain. Through the years, they have learned to cope with the feeling of disappointment and frustration. They rethink and try to come back with new proposals and different combinations of actions to relieve pain but sometimes they have to deal with the disappointment of not being able to solve the older adults’ problems. They continue to provide comfort and ease, but the pain remains.

*“…it’s tough when you don’t reach the goal, if you say so, but when you do, it feels good. I’ve done this, it is accomplished, treated and relieved and the pain is gone, BUT when the pain is constantly there all the time and when one meets all this despair,…it results in a sense of powerlessness for oneself, one doesn’t know…”*

They also deal with the knowledge that medications used to relief pain can potentially cause more harm than good in the older adults’ individual situation which adds to the sense of powerlessness. However, there is an ambition and effort to do good in the situation which contrasts to the sense of powerlessness.

### Striving to provide good health-care

The health care professionals meet the older adults in their own context and are able to address their problems and find ways to give help. The provision of health care to older adults with long-term pain at home is experienced as a continuous striving to provide good health care which is described as meaningful. There is an effort to do good which includes trying to relieve the pain. Sometimes it is difficult to deal with long-term pain that is settled in older adults’ bodies. The health care professionals describe with great satisfaction when they succeed in their effort to alleviate pain:

*“When you succeed, even if one does not succeed completely, small improvements make a big difference, and then you rejoice with the patient.”*

The health care professionals describe that they are striving to create trusting relationships; it gives a feeling of being important and needed. The striving to do good entails courage to listen and to represent the older adult in various situations which can be pictured as acting as the older adult’s lawyer. The striving to do good entails encouraging distraction of pain and focusing on things that give joy and meaning in life. They try to facilitate increased well-being by suggesting interventions that contribute to increased social interaction. The health care professionals adapt and direct their interventions and set concrete goals based on what is possible and important in the older adults’ daily lives. Examples of concrete goals are striving to be able to go to the grocery store or to take down the coffee tin from the bottom shelf in the cupboard. The caregivers have an overall aim to do good and increase the older adults’ quality in life, physical activity and independence.

*“… if one takes away at least a part of the problem with pain, one can improve quality of life and focus on the preconditions to maintain physical activity and independence and so. Even if they don’t become independent, it increases their comfort to not have as much pain.”*

The health care professionals experience that successful interventions give inner satisfaction and sense of meaningfulness for themselves. The striving to provide good health care can be understood through the health care professionals’ awareness of being task-focused and giving low priority to the dialogue even though they find it important. They have recognized that a visit at home can temporarily ease the older adult’s pain. The health care professionals’ experience that the phenomena of pain and the provision of health care for older adults living with pain is problematic and requires collaboration with other professions. The nurses, physiotherapists and occupational therapists work in teams in their effort to provide good health care. Collaboration also takes place with other care providers as assistant caregivers and social workers. The experience is that members in the team act based on their profession in order to provide good care. This entails distinguishing the problem and finding appropriate solutions. The importance of the team efforts to do good is illuminated by a health care professional as: *“It requires specific knowledge of pain and I don’t have that straight off, so one needs to work in a team.”* The provision of health care to older adults with long-term musculoskeletal pain is experienced as complex and the professionals in the team find that they complement each other which can be pictured as to *“add the puzzle together”*. They find strength in working together and being oriented towards striving to provide the best of care for the older adults. This effort requires understanding and respect for each and one of the older adults.

### Understanding and respect

Provision of health care to older adults with long-term pain is experienced to entail understanding and respect of the older adults’ pain, needs and desires in the situation. The understanding and respect comprise sensitiveness in knowing when the situation becomes unbearable, but also attention to the unspoken pain. The health care professionals try to balance older adults needs related to autonomy, integrity, independence and pain relief with attentiveness and sensitiveness The use of this approach in focusing on the older adults’ current problems is conveyed and expressed as, *“I’m very engaged and focused and empathetic”*. They analyze the situation and meet the person here and now in the moment. They try to offer suggestions, help the older adults make decisions for themselves, and to use trial and error to find the best solutions. They try to proceed cautiously, to be patient and to guide without intruding.

To provide health care to older adults living with long-term pain entails an understanding that the pain is multidimensional, e.g. physical, psychological, social and existential. The health care professionals describe that they try to encourage as much physical activity as feasible in the situation. There is an ambition to find a balance between activity and rest and to promote development of good body awareness. The health care professionals focus on understanding and development of the older adult’s knowledge of cause and inheritance of pain and how the body corresponds to movements, strain and pain. Sometimes they experience a reticence related to activities and receiving advice. Respect for this reticence is shown, since the older adults are autonomous and able to make their own decisions. Over time, the health care professionals develop security in their profession which make it easier to understand and respect rejection of given advice. This can be described in words as:

*“…when one was new in the profession, one had difficulties to accept that the offered help was not wanted. I think that when one is more secure in the profession and has more experience of life, one has it easier to accept that people have different priorities.”*

The health care professionals experience that providing health care requires an openness towards how the pain that is expressed and dealt with in various ways which is based on aspects as the older adult’s characteristics, ability to express oneself and cultural background. The health care professionals respect the older adults’ knowledge about appropriateness of different methods to alleviate pain in daily living and their experiences of how they can be used at home. This encompasses an ability to understand and respect that a method such as TENS is rejected due to the fact that the intervention and the health care providers that carry out the intervention might disrupt the older adult’s sense of “being at home”.

*“The pain does not disrupt so much, but if the personnel would come and one has to exercise all of a sudden, their daily living would be disrupted in a way they don’t want.”*

Provision of health care to older adults with long-term pain at home requires attentiveness and openness towards the person as a whole. This requires understanding and respect for the older adults’ needs and a striving to provide good health care despite a sense of powerlessness in the situation.

## Discussion

The significance of being able to remain in the encounter despite feelings of insecurity and uncertainty as a health care professional was highlighted in the findings. There was an overall awareness of the need to transfer one’s orientation from “doing” to “being”, something that was challenging and required courage. The effort to focus on the older adults’ needs in the overall life situation instead of the health problem itself has been argued by researchers [39, 44, 50] with the aim of supporting the older adults overall sense of health and well-being.

The findings described in the constituents: *striving to provide good health care* and *understanding and respect* implies that the health care professionals have a desire to provide person-centered care (c.f. [48]), despite lack of time. This can be understood as being grounded in the assumption that the human being is viewed as a whole (c.f. [36–38]). The goal of a relationship in which the health care professional learned to know the older adult was explicit in the findings and can be viewed in light of the concept “knowing the patient” [42–45] and Todres, Galvin and Dahlberg [40] reach towards “insiderness”. The findings show that the provision of health care is complex, challenging and requires courage. It demands the use of self through “reflective openheartedness” and knowledge of lifeworld-led care (c.f. [40]). Bindels, Cox, Widdershoven, van Schayck and Abma [64] argue the need of giving priority to a trusting relationship in the provision of care for community-dwelling frail older adults which also is highlighted in the findings in this study.

The constituent, *Sense of powerlessness* entailed feelings of frustration, inadequacy and hopelessness. One potential cause for the experienced lack of attentiveness might be the prevailing opinion among both health care providers, older adults and significant others that pain is a natural part of the aging process [25, 65–70]. Another potential cause that adds to the sense of powerlessness might be the existence of low priorities and negative attitudes in society related to provision of health care to older adults [31–34]. Issues about interventions as analgesics are an additional source for sense of powerlessness. The findings show that the health care professionals often end up in conflict between differences in demands and expectations from the older adults, other care givers and relatives. The tendency for a stoic attitude with a hesitance to report pain among older adults can in part explain that the older adults themselves express less worry about their situation than the relatives or other health care providers (c.f. [19, 28, 29, 71]).

The willingness to do good and the demand for understanding and respect, despite a sense of powerlessness, requires reflection and support. Continuous supervision sessions in groups can support health care providers who are dealing with older adults’ physical, psychological, social needs and existential anxiety but also support the health care providers’ feelings and shortcomings (c.f. [72–75]). Supervision can be a way for health care providers to learn and support each other in being open to older adult’s lifeworld, which encompasses the person as a whole as well as the situation (c.f. [39, 44]). It is stressed in the findings that it is important to work in teams to *“add the puzzle together”* and do the best in the situation for the older adult. However, the health care professionals experience a lack of time in the provision of health care. Research shows that working in teams not only improves the quality of care and is cost-effective, but also improves health care providers’ experiences of satisfaction and effectiveness [76]. Supervision in groups can be a way to support health care providers in tailoring and providing person-centred care. The supervision not only promotes learning from each other. It might also have a positive influence on the prevailing attitudes found in society in regard to working with older adults (c.f. [31, 35]).

The increasing number of older adults living at home with health problems such as long-term musculoskeletal pain is a challenge for those working in the home care and for society at large [9, 77–79]. However, previous research shows that older adults felt forced into learning to live with pain on their own [18, 19]. This stresses the importance of an individualized and holistically tailored care in the older adult’s home, care that takes into account the whole situation [55, 71].

### Methodological considerations

Qualitative interviews grounded in RLR [56] were chosen to collect data. This method was relevant in relation to the aim of capturing the participants’ experiences of the phenomenon. These experiences are valuable since research indicates low priority and negative attitudes (c.f. [31–34]) related to provision of health care to older adults. It is also important to raise the awareness of the predominance of long-term musculoskeletal pain among the increasing number of older adults due to the fact that this health problem often is unrecognized, under reported and under treated [21–24, 80]. This qualitative study has the advantage of being interdisciplinary (nurses, physiotherapist, occupational therapists) which is consistent with the orientation within home health care of today. The variation in profession and gender contributes various perspectives and facilitates data that is rich in nuances of qualitative meanings. However, profession and gender have not been acknowledged in the findings since the focus was the phenomenon itself as appropriate is in RLR (c.f. [56]).

There are variations in opinions about transferability and generalizations of findings in qualitative research. Dahlberg, Dahlberg and Nyström [56] state that the essence in a phenomenological research approach can be generalized in similar contexts. Stake [81] argue that the potential for qualitative findings to be transferred is determined by the reader since it depends on how the findings can be related to the readers’ context, knowledge and earlier experiences*.* Both these arguments support the findings which can be taken into account in the discussions and planning of education, development of individualized and holistic care to guide, and support for older adults living at home with musculoskeletal long-term pain. These findings can also be used as reference points in future and existing research findings in similar or various contexts.

## Conclusions

The findings indicate that the health care professionals had the ambition to do good and tried to provide health care that was holistic and sensitive to older adults’ needs. However, there was a significant sense of powerlessness in the situation. These findings address and support the need to develop methods that can be used as tools to guide and support older adults and health care providers in the selected context.

The method Reflective STRENGTH-Giving Dialogue was developed to meet this need [82] and this innovation needs be tested in a study. The intervention will consist of an educational program and continuous supervision to health care professionals (nurses/district nurses, physiotherapists, occupational therapists) as they accomplish reflective STRENGTH-Giving Dialogues with community dwelling older adults once a week. A tactful and challenging approach will be used by the health care professional to increase the person’s awareness of possibilities and choices in life. Furthermore, there will be a focus on joy and meaning in the older adult’s life in aim to support sense of strength, courage and well-being in daily living with pain.
